# Disruptions to U.S. local public health’s role in population-based substance use prevention and response during COVID-19

**DOI:** 10.1186/s13011-022-00499-7

**Published:** 2022-11-07

**Authors:** Kellie Hall, Francis Higgins, Karla Feeser Beach, Kabaye Diriba, Mandy Sladky, Timothy C. McCall

**Affiliations:** 1 National Association of County and City Health Officials, Washington, DC USA; 2 Mathematica Policy Research, Washington, DC, USA; 3 Substance Abuse and Mental Health Services Administration, Rockville, MD, USA; 4 Public Health – Seattle & King County, Seattle, WA, USA; 5 Department of Clinical Research & Leadership, School of Medicine and Health Sciences, The George Washington University, Washington, DC, USA

**Keywords:** Local public health, Overdose prevention, COVID-19

## Abstract

**Background:**

COVID-19 dramatically limited the scale and scope of local health department (LHD) work, redirecting resources to the response. However, the need for essential public health services—including substance use prevention—was not reduced.

**Methods:**

We examined six quantitative data sources, collected between 2016 and 2021, to explore the impact of the COVID-19 pandemic on LHD substance use-related services.

**Results:**

Before the pandemic, the proportion of LHDs providing some level of substance use prevention services was increasing, and many were expanding their level of provision. During the pandemic, 65% of LHDs reduced their level of substance use-related service provision, but the proportion of LHDs providing some level of services remained steady from prior to COVID-19.

**Conclusion:**

We discuss policy recommendations to mitigate the risk of service disruptions during future public health emergencies, including direct and flexible funding for LHDs and federal directives declaring substance use prevention services as essential.

## Background

The COVID-19 emergency has tested the public health, medical, and public safety infrastructure of the United States (U.S.) in unprecedented ways. As the focal point for public health in their communities, local health departments (LHDs) were particularly impacted by the pandemic’s onset. The immediate public health response required LHDs to shift staff rapidly and launch new services, including testing, contact tracing, and, eventually, vaccination drives. Much of this was accomplished in especially difficult circumstances. Like many other frontline workers, LHD staff confronted an increased workload and workplace safety concerns related to the virus. [1] Furthermore, widespread and politically motivated harassment to public health workers across the U.S.—as the faces of sweeping mitigation measures aimed at limiting the spread of COVID-19—compounded these challenges. [2]

Despite the dramatic shift in the scale and scope of LHD work, the need for ongoing delivery of essential public health services was not reduced. Of particular note is the work to reduce and respond to substance use, including the associated risk of unintentional drug overdoses. In one survey conducted in the U.S. early in the pandemic with 1,079 people with substance use disorder, 20% of respondents reported an increase in substance use and 14% reported being unable to access treatment and recovery services. [3] Difficulty accessing these services resulted in adverse emotional outcomes at a higher rate than respondents who did not report disruptions (87% vs. 72%). [3] As substance use was increasing, access to treatment and recovery services was declining.

This is particularly concerning for drug use as overdoses were already a major crisis in the years leading up to the pandemic. In 2019, overdose death tolls surpassed 70,000 people and rose drastically in 2021, totaling over 100,000. [4] Data collected in the U.S. since the onset of the pandemic indicates that the increased overdose burden was not evenly distributed, with one study noting that the Black-white overdose mortality gap rose from 4.8 to 100,000 in 2019 to 9.9 by the end of 2020. [5] Another study reported that, after holding steady with the white population for many years, the overdose rate among American Indian and Pacific Islanders (AAPI) rapidly increased during the pandemic and now represents the highest single population overdose mortality rate in the country. [6] Overdose deaths among people experiencing homelessness in Los Angeles County also rose by 33% in 2020 as compared to the previous year [7]—a dangerous increase for a population already experiencing difficulties in accessing regular care. [8]

There are many potential and interrelated forces that could have contributed to this trend, such as changes in the drug supply or social isolation exacerbated by pandemic safety measures. An environmental scan of the impact on harm reduction services found that the pandemic caused supply chain issues and decreased staff capacity among harm reduction organizations in the U.S. [9] Further studies examining the link between the pandemic and rising overdose numbers note that public health staff reductions or reallocation may have played a causal role in increasing the risk of overdose in the U.S. and British Columbia. [10] One study in the journal *AIDS and Behavior* found that 25% of SSPs in the U.S. reported closures in the initial month after the onset of the pandemic; however, respondents in this study also noted that innovations in service delivery allowed SSPs to overcome these early barriers. [11] Another found that buprenorphine prescriptions dropped in the initial months of the pandemic, though they had generally rebounded to pre-pandemic levels by August 2020. [12]

Despite the significant measured impact of COVID-19 on substance use outcomes, the literature exploring the virus’ impact on substance use prevention and response services in the U.S. is limited. Much of the relevant literature that exists focuses on changes in access to substance use-related services during the pandemic, but little research has been published about the prevalence of service disruptions. As access to services can be impacted by other factors unrelated to service disruptions, such as access to transportation, it is important to investigate the breadth of changes to substance use prevention and response services. Evidenced-based prevention and treatment services are known to improve outcomes for people with substance use disorder, so any changes to their provision could contribute to the worsening outcomes reported since the onset of COVID-19.

### Case study

The National Association of County and City Health Officials (NACCHO) frequently hears from partner LHDs about COVID-19 impacts to communities, and several of the findings of the environmental scan mentioned above are aligned with their experiences. One such partner, Public Health – Seattle & King County (PHSKC), is particularly notable as the Seattle area was the initial documented entry point for COVID-19 into the U.S. [13] The pandemic impacted PHSKC’s delivery of substance use prevention and treatment programs—including those that specifically serve people who use drugs—in a major way. For example, key staff were re-assigned to respond to the pandemic. This included the Overdose Prevention and Response Team’s epidemiologist and communications specialist, which limited capacity to access and interpret substance use data, as well as to disseminate key messages to the public that could prevent unintentional overdoses.

Two programs particularly affected were the SSP and low barrier buprenorphine clinic. While these services did not have any closures—as core functions of local public health—service delivery changed substantially. The SSP locked its doors and allowed only one client inside at a time for a brief service interaction; as of July 2022, only two people are allowed entry. Staff report this social distancing requirement of the COVID-19 pandemic resulted in considerably less information sharing with clients on key prevention topics, including changes in the drug supply, and in less client engagement in other related onsite services (e.g., hepatitis C testing and treatment). To reduce the number of visits by clients and reduce risk of COVID-19 exposure, the SSP switched to a needs-based distribution approach, in alignment with CDC’s endorsement of needs-based distribution. [14] King County’s Pathways program, a low barrier buprenorphine clinic, adapted to the pandemic by limiting in-person visits and conducting phone visits whenever possible. This telehealth approach made it easier for some patients to access care but created challenges for others, including those with limited access to devices, severe mental illness, and those who otherwise struggle with technology or remote communication.

In PHSKC’s jurisdiction, service disruptions coincided with a dramatic increase in the availability of fentanyl and, consequently, an estimated 350% increase in fatal overdoses involving fentanyl between 2019 and 2021. [15] With fewer supports and limited access to services across the county, particularly for the most marginalized, staff heard countless stories of people in recovery returning to substance use during the stress of the pandemic. Service disruptions in the area remain, largely due to staffing shortages and funding constraints, putting ongoing stress on both patients as well as providers who often have nowhere to refer them. One staff described the behavioral health system as “decimated.”

### Purpose

No comprehensive literature exists that examines the impact of the COVID-19 pandemic on the provision of substance misuse prevention and response services by LHDs. As the key hub for public health services in their community, LHD capacity to provide services plays an outsized role in combatting the ongoing overdose epidemic. This paper seeks to explore both the impact of the pandemic on LHD substance use prevention and response services, as well as their initial response to service disruptions.

## Methods

### Data collection

All data were collected by NACCHO via six cross-sectional online surveys between 2016 and 2021 (Table 1). The six surveys included the 2016 National Profile of Local Health Departments study (RR = 76%), the 2018 Forces of Change survey (RR = 61%), the 2019 National Profile of Local Health Departments study (RR = 61%), the 2019 Injury and Violence Prevention survey (RR = 51%), the 2020 Forces of Change survey (RR = 29%), and the 2021 Injury and Violence Prevention survey (RR = 26%). For each of these surveys, the unit of analysis is organization, so each LHD provided one response to the survey. These data collection efforts were part of existing longitudinal studies beginning prior to the onset of the COVID-19 pandemic and spanning the first year of the pandemic.

**Table 1:** Data sources, survey sample, and dates of data collection

Data source	Responses	Dates of data collection	Pandemic phase
2016 National Profile of Local Health Departments study	Census of all LHDs	January to April 2016	Pre-COVID-19
2018 Forces of Change survey	Representative sample of LHDs	March to May 2018	
2019 Injury and Violence Prevention survey	Sample of LHDs that responded to 2018 Forces of Change and reported conducting opioid use and abuse activities in the prior year	January to February 2019	
2019 National Profile of Local Health Departments study	Census of all LHDs	March to August 2019	
2020 Forces of Change survey	Representative sample of LHDs	October 2020 to March 2021	Mid-COVID-19
2021 Injury and Violence Prevention survey	Representative sample of LHDs	March to May 2021	

***National Profile of Local Health Departments (Profile) study***^1^. The Profile study (2016, 2019) assesses LHD infrastructure and practice by describing how funding, staffing, governance, and activities of LHDs vary across the U.S. Every three years, the survey includes a set of core questions sent to a census of LHDs in the U.S. and two module questionnaires sent to statistically representative samples of all LHDs, stratified by size of population served. In 2016, the Profile study (bit.ly/3g2eTml) was distributed online to a total of 2,533 LHDs from January to April 2016 (n = 1,930; 76% response rate). Two states (i.e., Hawaii and Rhode Island) were excluded from the study because they had no LHDs. [16] In 2019, the Profile study (bit.ly/3VmhqYo) was distributed online to a total of 2,459 LHDs from March to August 2019 (n = 1,496; 61% response rate). Rhode Island was excluded from the study because the state has no LHDs. [17]

***Forces of Change surveys***^2^. The Forces of Change survey (2018, 2020) assesses changes in LHD capacity and practice driven by emergent issues in public health. Each year, the survey is sent to a statistically representative sample of LHDs, stratified by size of population served and state. Because LHDs with large population sizes represent a relatively small portion of all LHDs, these LHDs were oversampled to ensure enough responses for the analysis, which used weighting to adjust for this oversampling and differential response rates between jurisdiction sizes (see “statistical analysis”). The 2018 Forces of Change survey (bit.ly/3RSP8So) was distributed online to a statistically representative sample of 966 LHDs from March to May 2018 (n = 591; 61% response rate). Two states (i.e., Hawaii and Rhode Island) were excluded from the study because they had no LHDs. [18] The 2020 Forces of Change survey (bit.ly/3ChwNZC) was distributed online, and a module with the set of questions relevant to this paper was sent to a statistically representative sample of 905 LHDs from October 2020 to March 2021 (n = 236; 26% response rate). Rhode Island was excluded from the study because they have no LHDs; Florida was also excluded from the study at the request of the state department of health. [19]

***Injury and Violence Prevention (IVP) surveys***^3^. The IVP survey (2019, 2021) assesses LHD capacity, infrastructure, and practice in addressing opioid and other drug use. From January to February 2019, the survey (bit.ly/3T7XhmY) was distributed online to 388 LHDs that responded to the 2018 Forces of Change and reported conducting activities to address “opioid use and abuse” at any time in the prior calendar year (n = 198; 51% response rate). [20] In 2021, the survey (bit.ly/3eqilqf) was sent to a statistically representative sample of 766 LHDs, stratified by 2 variables: jurisdiction size and Census region. The questionnaire was distributed to from March to May 2021 (n = 196; 26% response rate). [21]

### Statistical analysis

Data were managed and analyzed in Stata 15.1 (StataCorp, College Station, TX). While the surveys were distributed to nationally representative samples, differing response rates required adjustments to ensure the final respondent samples were statistically representative in line with the sampling method. Nationally representative estimates from the Profile studies and the Forces of Change surveys were computed using survey weights to be representative of various jurisdiction sizes in the U.S., such that responses were weighted proportionally to their distribution nationwide. Responses from the 2019 IVP survey were computed without weights because they were collected from a sample of LHDs identified as having conducted relevant activities via the 2018 Forces of Change survey; no census-level data was available to use in computing weights. Nationally representative estimates from the 2021 IVP survey were computed using survey weights to be representative of various jurisdiction sizes and geography (Census region) in the U.S., such that LHDs within a region and jurisdiction size were weighted proportionally to their distribution nationwide. Descriptive statistics and confidence intervals for each survey were generated to examine trends over time; where comparisons between years were made, p-values from one-sample proportions tests were computed to make cross-year comparisons and determine significant differences between samples and timepoints.

## Results

### Pre-COVID-19 context (2016 and 2019 Profile studies, 2019 IVP survey)

**Fig. 1:**
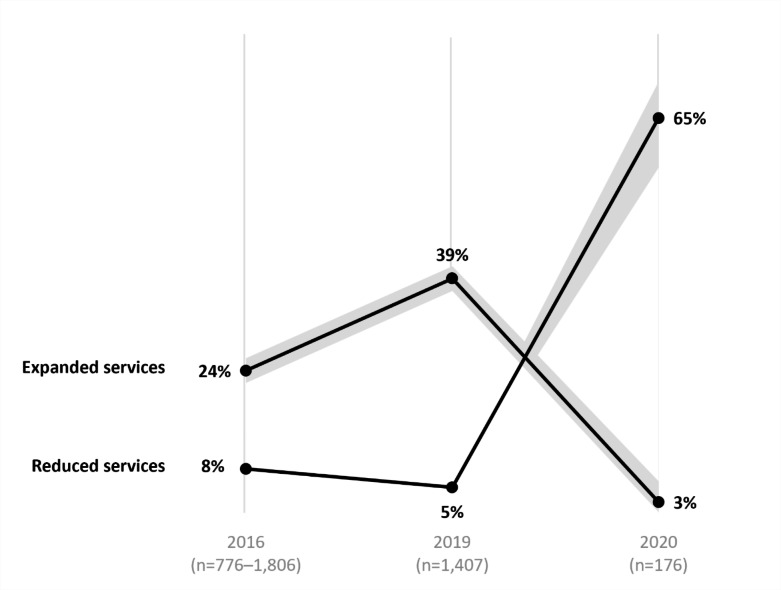
Expansion or reduction in level of substance use service provision over time. Data presented by proportion (%) of LHD respondents. Shading represents 95% confidence intervals. LHDs reporting “no change” in service provision are not displayed

Prior to the emergence of COVID-19, LHDs were increasingly supporting work to prevent and respond to substance use disorder. Data from the 2016 and 2019 Profile studies revealed that the proportion of LHDs directly providing population-based substance use prevention services increased slightly (76–83%; p < .01). In addition, the proportion of LHDs expanding their provision of these substance use prevention services increased (24–39%; p < .001) (Fig. 1). Finally, results from the 2019 IVP survey revealed that opioid-related staffing was trending up prior to the pandemic; among LHDs that conducted activities addressing opioid use in the prior year, 40% reported that opioid-related staffing increased, while only 3% reported a decrease from the prior year.

### Impact of COVID-19 (2020 forces of Change survey, 2021 IVP survey)

Data from the 2020 Forces of Change survey show that almost two in three LHDs (65%) reduced their provision of substance use services—a significant difference compared to before the COVID-19 pandemic (Fig. 1). Despite these widespread reductions in service provision, data from the 2021 IVP survey revealed that a majority of LHDs (66%) continued to engage in work to prevent and respond to drug use (measured dichotomously: yes or no), and this was comparable to before the pandemic (65%, measured in 2018 Forces of Change survey). In other words, while almost two in three LHDs reduced their provision of substance-use related services during the pandemic, LHDs providing some level of services remained steady from prior to the pandemic.

Although LHDs offered some form of substance use prevention and response services during the pandemic, less than one quarter of LHDs addressed opioid use (24%) or alcohol use (23%) in their COVID-19 response efforts, as reported in the 2020 Forces of Change survey. In addition, 47% of LHDs reported in this survey that they addressed populations with depression or other mental health conditions, and fewer than a quarter developed COVID-19 messaging for populations with mental health (25%) or substance use (19%) disorders. Over four in five LHD respondents to the 2020 Forces of Change survey (82%) reported reassigning staff from regular duties to support COVID-19 activities, and 15% specifically reassigned staff from IVP programs.

**Fig. 2:**
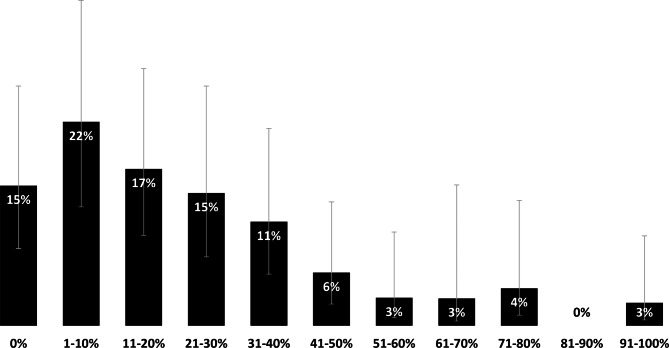
Estimated percent increase in overdoses in 2020 compared to 2019. Note the estimated percent increases may have been obtained from subjective assessments made by LHDs. Data presented by proportion (%) of LHD respondents (n = 93). Bars represent 95% confidence intervals

Over one in seven LHD respondents to the 2021 IVP survey (15%) reported that their staffing to address drug overdoses, specifically, decreased in 2021; 95% of these LHDs indicated the decrease was a result of the COVID-19 response. However, very few LHDs reported in the 2019 IVP survey eliminating drug overdose-specific services (between 0% and 5%) (Table 2).^4^ All services were more likely to be modified for the virtual environment (e.g., telehealth) than ended. Simultaneously, 62% of LHDs responding to the 2021 IVP survey self-estimated that their jurisdiction experienced a more than 10% increase in fatal and nonfatal overdoses in 2020 compared to 2019 (Median: 11–20%, Mode: 1–10%; Fig. 2).^5^

**Table 2:** Changes to substance use services resulting from the COVID-19 pandemic and associated safety regulations^6^

	Service provision was modified Weighted % [95% CI]	Service was limited Weighted % [95% CI]	Service ended Weighted % [95% CI]	COVID-19 did not affect provision Weighted % [95% CI]	LHD did not provide service prior to COVID-19 Weighted % [95% CI]	n
Family counseling	7 [4, 11]	3 [1, 6]	0 [0, 0]	1 [0, 7]	90 [84, 93]	185
Peer navigation	11 [7, 16]	1 [0, 0.3]	1 [0, 4]	4 [2, 9]	83 [77, 88]	187
Detox programs	4 [2, 8]	0 [0, 1]	0 [0, 0]	1 [0, 4]	94 [90, 97]	183
Fentanyl testing	5 [3, 9]	2 [1, 6]	0 [0, 0]	0 [0, 1]	92 [88, 95]	184
Syringe service programs	8 [5, 12]	6 [3, 11]	0 [0, 0]	2 [1, 5]	85 [79, 89]	185
Overdose response teams	8 [5, 13]	5 [3, 10]	1 [0, 4]	2 [1, 6]	84 [77, 88]	184
Naloxone distribution, education, and/or training	17 [12, 23]	12 [8, 18]	2 [1, 5]	9 [5, 15]	60 [52, 67]	187
Peer support groups	2 [1, 6]	1 [0, 3]	0 [0, 3]	2 [1, 6]	95 [90, 97]	187
Neonatal abstinence syndrome services	7 [4, 11]	3 [1, 6]	0 [0, 0]	1 [0, 7]	90 [84, 93]	185
Linkages to care	23 [17, 29]	13 [9, 19]	1 [0, 4]	7 [4, 12]	56 [49, 64]	184
Medication for opioid use disorder: Buprenorphine, Methadone, and/or Naltrexone	4 [2, 8]	2 [1, 6]	0 [0, 0]	2 [1, 6]	91 [86, 95]	185
Community re-entry programs	4 [2, 8]	1 [1, 4]	0 [0, 0]	2 [1, 7]	93 [88, 96]	185
Community education and outreach	21 [16, 28]	20 [14, 26]	5 [2, 9]	4 [2, 8]	50 [43, 58]	187
Medication takeback events	7 [4, 12]	4 [2, 9]	4 [2, 9]	2 [1, 5]	82 [75, 87]	187
Academic detailing	3 [2, 7]	3 [2, 7]	1 [0, 3]	3 [1, 8]	89 [84, 93]	185
Housing assistance	4 [2, 7]	3 [2, 7]	0 [0, 0]	3 [1, 8]	90 [85, 94]	185
Medication for opioid use disorder: jail-based programs	2 [1, 6]	0 [0, 1]	0 [0, 1]	2 [1, 7]	95 [90, 97]	184
HIV/STI testing	14 [10, 20]	20 [15, 27]	1 [0, 4]	6 [3, 10]	59 [52, 67]	184
Anti-stigma campaigns	10 [6, 15]	12 [8, 18]	3 [1, 7]	7 [4, 11]	68 [61, 75]	186
Crisis hotline	2 [1, 5]	1 [0, 3]	0 [0, 0]	5 [2, 9]	93 [88, 96]	186
Syringe litter drop boxes	2 [1, 5]	5 [2, 9]	0 [0, 0]	7 [4, 11]	87 [81, 91]	184
Medication drop boxes	2 [1, 6]	4 [2, 8]	1 [0, 3]	9 [5, 14]	84 [78, 89]	187

## Discussion

Before the COVID-19 pandemic, the proportion of LHDs conducting substance use prevention and response activities was increasing, and many LHDs were expanding their level of provision of these services. During the pandemic, LHDs overwhelmingly reported reducing their level of service provision. However, only a few ended their services entirely, suggesting substance use is considered a core service or function by LHDs. Overall, the pandemic did not impact whether or not LHDs were providing these services; rather, it affected the degree to which they could provide them.

Underlying drivers of these substance use service disruptions include limited workforce capacity, shifts in staff priorities and roles, and unplanned modifications to service delivery. Services that require in-person contact (e.g., medication takeback events, academic detailing, community outreach, HIV/STI testing) were particularly impacted by lockdown and social distancing regulations. LHDs discontinued or reduced their level of provision of these services during the pandemic at the highest rates. On the other hand, LHDs were able to continue providing most services—particularly those that could easily be transitioned to a virtual or hybrid environment. However, maintaining services despite limited resources can have adverse impacts on workers. Many LHD staff were asked to complete both their regular duties and those to respond to COVID-19. [19] U.S. public health staff were stretched thin responding to the pandemic and experienced adverse mental and emotional outcomes, including stress and burnout [22]—all while working to support people with mental and behavioral health disorders.

These findings reflect trends in workforce and service provision elsewhere in the U.S. [23, 24] but are especially alarming given what is known about the effects of the pandemic and measures to contain it on behavioral health and substance use. The mental health costs associated with the fear and anxiety surrounding a pandemic and related mitigation measures is well documented. [25] In particular, COVID-19 lockdowns limited person-to-person contact, which has been shown to exacerbate adverse mental health outcomes for people with substance use disorder. [26] This shift also necessitated a transition in service delivery from in-person to virtual care, which can increase access to services for some (e.g., people without access to transportation) but not for others. One study found that Black and rural patients are less likely to receive buprenorphine via telemedicine compared to white and urban populations. [27] Furthermore, providing services via telehealth can also limit the quality care. For example, it is more difficult to achieve the peer-to-peer social connection that has been shown to improve outcomes associated with substance use recovery services in digital spaces compared to in-person. [28] Compounding these challenges, LHDs were stretched thin and lacked the capacity to conduct the robust surveillance needed to align substance use prevention messaging with these evolving changes in the substance use context.

While there was an increasing trend in drug overdoses before the pandemic, these findings highlight possible drivers of unprecedented increases during the pandemic, which disproportionately impacted people of color and rural communities. [29] At the same time, this demanding work environment led to innovations in substance use prevention that can be leveraged to ensure LHDs are prepared for the next public health emergency. In particular, there have been advances in non-person-to-person substance use prevention approaches, including the installation of vending machines with harm reduction supplies [30] and those outlined in the case study below. Finally, there has been a growth in distinct staff positions at LHDs that respond to overdoses, such as overdose prevention coordinators or harm reduction specialists. Continued support for these roles, along with sustained funding, are needed investments to minimize the impact of capacity that is redirected away from substance use services during public health emergencies.

### Study Limitations

Limitations of this study include that all data are self-reported and not independently verified. LHDs may have provided incomplete, imperfect, or inconsistent information for various reasons, including that no survey question required a response or that they were unsure of or unable to provide precise data. While the surveys include definitions for many items, not every item or term is defined. Consequently, respondents may have interpreted questions and items differently. In addition, the paper has a heavy emphasis on drug overdose prevention and response services; NACCHO did not collect similarly detailed data focused on alcohol, tobacco, or other substances. In addition, survey response rate varied by survey and some response rates (e.g., 2020 Forces of Change survey) were exceptionally low; while survey responses were weighted to balance responses by jurisdiction size and differing rates of non-response, nonresponse bias cannot be discounted, and these results should be interpreted with caution. In particular, given the drop in response rate from before the pandemic, it is possible that the two may be related and overburdened LHDs were less likely to respond; thus, the results presented here could underestimate service reductions during the pandemic.

## Conclusion

LHDs were making great advancements in the substance use space—and specifically, with the opioid overdose epidemic—pre-pandemic, but those advancements were stymied with the onset of COVID-19. Given the findings, it is important for federal agencies and national organizations to support LHDs in returning to pre-pandemic levels of substance use service provision through direct funding and technical assistance. Given the disproportionate negative impacts of service disruptions on marginalized populations, it is imperative that local public health works to ensure this availability of substance use treatment and resources for all those in need. With this in mind, support should focus on the circumstances of service provision in a post-pandemic world, especially as it relates to supporting telehealth for vulnerable and underserved populations. In addition, LHDs are not working in a vacuum to address substance use, so support should focus on cultivating effective multi-sector partnerships or coalitions.

Study findings also suggest the need for service protections during public health emergencies. For example, federal policies that deem substance use prevention services as essential would limit the reallocation of those resources to pandemic response. SSPs and Naloxone distribution would particularly benefit, with approximately one-third of LHDs reporting service limitations or closures during COVID-19. Finally, flexible funding provided directly to LHDs through federal and state sources is needed. A limited amount of LHD revenue is made up of direct funding, [16] and often, that investment is issue specific. [31] This prescriptive approach hinders strategic alignment of funding to address priority community needs and adapt work quickly to changing contexts. Instead, LHDs must focus on the one threat most prominent during a public health emergency (e.g., COVID-19, infectious disease, pandemic preparedness) in lieu of continuing and strengthening essential services.

## Data Availability

The datasets used and/or analyzed during the current study are available from the corresponding author on reasonable request.

## List of abbreviation

LHDs: Local health departments
U.S.: United States
CDC: U.S. Centers for Disease Control and Prevention
SSPs: Syringe services programs
PHSKC: Public Health – Seattle & King County
Profile: National Profile of Local Health Departments
IVP: Injury and violence prevention
NACCHO: National Association of County and City Health Officials
PHSKC: Public Health – Seattle & King County

## Footnotes

1 Funding was provided by the Centers for Disease Control and Prevention and the Robert Wood Johnson Foundation® in Princeton, New Jersey.

2 Funding was provided by the Centers for Disease Control and Prevention and the Robert Wood Johnson Foundation® in Princeton, New Jersey.

3 Funding was provided by the Centers for Disease Control and Prevention.

4 The survey question LHDs responded to was: “Were any of the following services provided directly or contracted out by your LHD affected by the COVID-19 pandemic and associated safety regulations?”

5 The survey question LHDs responded to was: “By approximately what percentage have overdoses increased in 2020 compared to 2019?”

6 The data in Table 2 describe self-reported changes in services by LHDs responding to the 2021 IVP survey. LHDs selected from the displayed options to identify whether the agency modified, limited, or ended their service provision.
